# Modern Biomarkers in IgA Nephropathy and Their Potential in Paediatric Research

**DOI:** 10.3390/jcm14093263

**Published:** 2025-05-07

**Authors:** Agnieszka Such-Gruchot, Małgorzata Mizerska-Wasiak, Emilia Płatos, Małgorzata Pańczyk-Tomaszewska

**Affiliations:** 1 Department of Paediatrics and Nephrology, Medical University of Warsaw, 02-091 Warsaw, Poland; agnieszkasuch.g@gmail.com (A.S.-G.); mpanczyk1@wum.edu.pl (M.P.-T.); 2 Student’s Scientific Group at the Department of Paediatrics and Nephrology, Medical University of Warsaw, 02-091 Warsaw, Poland; emiliaplatos@gmail.com

**Keywords:** IgA nephropathy, children, biomarkers

## Abstract

Immunoglobulin A nephropathy (IgAN) is one of the most common forms of glomerulonephritis and one of the main causes of end-stage renal disease (ESRD), affecting up to 40% of patients after 20 years of the disease. Over the past few years, several studies have been conducted to search for biomarkers that can diagnose IgAN in a non-invasive way, select patients in the asymptomatic phase, assess the progression of the disease process and monitor its efficacy, and assess the risk of IgA nephropathy progression. Non-invasive investigations using molecules as an alternative to renal biopsy have potential relevance for diagnosis, the determination of treatment efficacy and the assessment of disease activity. For the early screening of IgAN with isolated haematuria, these factors may be useful for early intervention and result in a reduction in the risk of impaired renal function. The clinical studies discussed in this review underline that featured molecules show potential as biomarkers for the diagnosis and assessment of disease progress in IgAN.

## 1. Introduction

Immunoglobulin A nephropathy (IgAN), described for the first time in 1968 by Berger and Hinglais [1], is considered to be one of the most common forms of glomerulonephritis and one of the main causes of end-stage renal disease (ESRD), which occurs due to damage to the nephrons by the accumulation of immunoglobulin A (IgA) deposits [2,3]. The incidence of ESRD reaches up to 40% of patients after 20 years of the disease, which can shorten life expectancy by about 10 years [4]. The prevalence of the disease varies according to latitude. IgAN is more prevalent in Asian and Pacific regions, where it causes 60% of primary glomerulopathies. It is responsible for 30% and 10% of cases of glomerulonephritis in Europe and America, respectively [5]. The overall incidence of IgAN in the population is at least 2.5/100,000/year. In the population of children, depending on the data source, the incidence rate fluctuates between 0.57/100,000/year (Europe and the USA) and 4.5/100,000/year (Japan) [2]. In a study evaluating the paediatric population of six European countries, it was 0.2 per 100,000 children per year [6].

IgAN is characterised by a variety of clinical manifestations and variable pathomorphological changes. The presence of IgA deposits in the glomerular mesangium over other deposits is the condition for the diagnosis; in addition, mesangial proliferation may occur [7]. Clinical manifestations include isolated microscopic or macroscopic haematuria, often with concomitant proteinuria, or arterial hypertension, and in the most severe cases, they take the form of rapidly progressive glomerulonephritis (RPGN).

More than 50% of patients with IgAN are diagnosed after an incidental finding of microscopic haematuria, arterial hypertension, proteinuria, or reduced glomerular filtration rate (GFR) [7,8]. The only method for the diagnosis of IgAN is renal biopsy. Moreover, immunofluorescence (IF) shows dominant IgA deposits in the glomerular mesangium, and routine histopathology demonstrates active and chronic changes in glomeruli and renal interstitium [9]. The spectrum of renal changes varies, ranging from undetectable or small to diffuse proliferative lesions with crescents [10]. The main aim of the renal biopsy is the diagnosis of the disease. Furthermore, this technique determines whether the pathological process is active and thus potentially reversible, allowing the progression of chronic lesions to be assessed. Oxford classification (MEST-C) uses renal biopsy evaluation to predict the outcome in IgAN [10]. Kidney biopsy, as an invasive method, is associated with a risk of complications. Additionally, based on the evaluation of renal biopsy, we can only assess the severity of IgAN at the time of the examination, without being able to monitor the disease process further. It seems, therefore, extremely important from a clinical perspective to look for molecular, genetic or biochemical markers that can replace biopsy.

Nowadays, biomarkers applied in medicine, thanks to their measurable biological features determined by a specific process or physiological condition, are used for non-invasive and precise diagnosis. A biomarker can be a molecule present in body fluids, including urine, serum or tissues. A biomarker is considered to be an objectively measurable biological indicator that can point to the presence of a disorder and also control the body’s response to therapeutic interventions.

Over the past few years, several studies have been conducted to search for biomarkers that can diagnose IgAN in a non-invasive way, select patients in the asymptomatic phase, assess the progression of the disease process and monitor its efficacy, and assess the risk of IgA nephropathy progression.

## 2. Pathogenesis and Biomarkers

IgAN is an immune-mediated inflammatory disorder. Its pathophysiology is associated with the overproduction of abnormally glycosylated IgA as a response to infections, most commonly of the upper respiratory tract [11,12]. Although the factors underlying the pathogenesis of IgAN remain unclear, the cause of its development is assumed to be explained by the four-hit hypothesis, also known as the multi-hit theory, which has been mentioned in high-quality studies. The multi-hit theory suggests the production of IgA1 with galactose insufficiency (Gd-IgA1; Hit 1), IgG or IgA autoantibodies, which recognise Gd-IgA1 (Hit 2), followed by the formation of immune complexes (Hit 3), and the accumulation of these complexes in the glomerular mesangium (Hit 4), also modified by genetic or environmental factors.

A hallmark of this disease is an abnormal glycosylation of the hinge region of the IgA1 molecule, leading to a reduction in the amount of galactose attached to it. These antibodies are described as galactose-deficient (Gd-IgA1). Their concentration in the serum of patients with IgA nephropathy is significantly higher than in healthy individuals [13]. GdIgA1 molecules can form polymers. Specific IgA and/or IgG antibodies are produced to target the abnormal IgA1 molecule, which fuse together to form IgG-IgA1 immune complexes in the blood, preventing their degradation in the liver by the asialoglycoprotein receptor (ASGPR). Higher concentrations of IgG antibodies in the oligosaccharide region were reported in patients with IgAN [14].

Thereafter, immune complexes are deposited in the renal mesangium, and the IgA1 particle binds to transferrin receptors (CD71) in the hinge region [15].

The accumulation of the complexes in the mesangium leads to the activation of complement pathways and induces a chronic immune-mediated inflammatory process. It plays an important role in the pathogenesis and progression of IgAN. Complement activation occurs via alternative and lectin pathways [16].

The activation of the lectin pathway involves the mannose-binding lectin (MBL) and ficolins, which, through the activation of MBL-related serine proteases such as MASP-2, induce the activation of C4 and C2 and the production of the C4bC2a-C3-convertase complex. This mechanism may play an important role in complement activation by apoptotic or necrotic tissue and enhance renal injury mediated by IgA [17].

**Figure 1:**
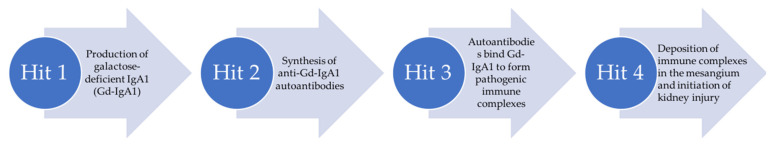
Multi-hit theory.

An increased production of cytokines and growth factors leads to cell and mesangial matrix proliferation. The chronic inflammatory process eventually leads to the fibrosis of the renal interstitium, the main mechanism responsible for renal failure in this case [16,18]. (Figure 1).

## 3. Gd-IgA1

The establishment of an immune complex with Gd-IgA1 is an important factor in the pathogenesis of IgAN. High serum GdIgA1 antibody levels are associated with a risk of disease activity [19].

According to Kiryluk K et al., serum levels of abnormally glycosylated IgA1 in patients with IgAN were higher than in healthy individuals or those with other kidney diseases [20].

In a study by Suzuki Y et al., higher levels of GdIgA1 and IgA/IgG immune complexes were linked to increased haematuria and proteinuria, which suggests that the concentration of the molecule is correlated with the severity of disease activity [19].

In a Poland-based study involving children with IgAN and IgA-associated vasculitis nephropathy (IgAVN), a positive correlation was observed between Gd-IgA1 levels and increasing serum creatinine levels in patients with IgAN, indicating a possible prognostic value for monitoring disease progression [21].

Gd-IgA1 levels can thus be considered a potential marker of IgAN progression in children.

## 4. Anti Gd-IgA1

IgAN is characterised by immune complexes consisting of Gd-IgA1 and an IgG/IgA antibody targeting the molecule. Gd-IgA1 is recognised by glycan-specific antibodies of IgG or IgA1 isotypes in IgAN patients and healthy children. The binding of these immune complexes to mesangial cells induces renal symptoms specific to IgAN.

William J. Placzek et al. evaluated the association between serum Gd-IgA1 levels and IgG or IgA serum Gd-IgA1-specific autoantibodies from 135 adult patients with histopathologically confirmed IgA nephropathy, 76 patients with other renal diseases and 106 healthy individuals [22]. An analysis of the results demonstrated a positive correlation between serum IgG autoantibody concentrations in patients with IgA nephropathy in comparison to the control group (patients with other kidney diseases and a healthy population) [22]. Moreover, the evidence suggested that IgG is the dominant isotype of Gd-IgA1-specific antibodies in IgAN [22]. A similar study conducted by Berthoux et al. shows that the concentration of IgG and IgA autoantibodies in serum was crucial in patients with IgAN (assays performed at the time of renal biopsy) in contradiction to healthy subjects and patients with renal disease other than IgAN, indicating the involvement of these antibodies in the development of IgAN [23].

According to Bellur SS et al., the concentration of IgG antibodies specifically targeting Gd-IgA1 correlates with the severity of IgAN and proteinuria [24].

Hitoschi Suzuki et al. proved that elevated levels of anti-Gd-IgA1 antibody in the serum of patients with IgAN were associated with proteinuria [18].

The following study demonstrates that anti Gd-IgA1 antibodies are linked to IgAN pathophysiology and may be a specific marker for this disease and a potential therapeutic target.

## 5. IgA/C3

Serum C3 complement levels in patients with IgAN are usually normal, while serum IgA1 and IgA-related immune complexes are elevated [11]. Serum IgA in patients with IgAN is significantly higher than in patients with other types of nephropathies, and the serum IgA/C3 ratio appears to be a more reliable marker than isolated IgA [25].

The serum IgA/C3 ratio is generally considered to be a marker of progression in IgAN. Zhang Jun et al. studied the IgA/C3 ratio in 217 patients with IgAN, identifying disease progression in 9.7% of patients with higher values of the ratio [25].

Komatsu et al. confirmed that a high serum IgA/C3 ratio (>4.5) in patients with IgAN correlated with poorer renal outcomes among Japanese patients [26].

Wen-yu Gong et al. assessed the diagnostic value of the serum IgA/C3 ratio among 1095 patients with primary glomerular nephropathy in China [27]. The investigation found that the IgA/C3 ratio in the group of patients with IgAN was significantly higher than in the group without IgAN; however, there was no correlation between the ratio and the severity of proteinuria or the progression of chronic kidney disease [27].

The IgA/C3 ratio has also been investigated in the paediatric population. Mizerska-Wasiak et al. evaluated a group of 55 children with IgAN [28]. They found no significant association between the IgA/C3 ratio at disease onset and reduced GFR or persistent proteinuria at long-term follow-up. These data suggest that the IgA/C3 ratio is not a good indicator of progression of IgAN in childhood [28].

Mizerska-Wasiak et al. also analysed the association of the IgA/C3 ratio with the histopathological features of the renal biopsy assessed using the Oxford classification in a group of 89 paediatric patients with IgAN. The mean values of the IgA/C3 ratio were significantly higher in patients with M1, S1 and T1 compared to M0, S0 and T0, respectively. The IgA/C3 ratio in children with IgAN may be a useful marker of the severity of lesions on renal biopsy assessed using the Oxford classification [29].

## 6. Complement C4

Complement components, as well as complement regulatory factors determined in renal biopsy, urine and serum, may be valuable biomarkers for the evaluation of complement system activation and the prognosis of patients with IgAN.

The concentration of the C4 component of the complement system has been investigated several times over the past few years.

Bi T.D. et al. presented the results of a retrospective study in a group of 1356 patients with primary IgAN, in which they evaluated the association between serum C4 levels and clinical manifestations and their prognosis [30]. They found a clear correlation between plasma C4 concentration and clinical risk factors for nephropathy. C4 levels were positively related to daily urinary protein excretion, IgA and C3 levels but negatively correlated with the glomerular filtration rate and serum albumin. Additionally, they observed that C4 levels increased with the exacerbation of tubulointerstitial damage, crescent count and glomerular hyalinosis rate [30].

Although complement activation is known to be involved in the pathogenesis of IgAN, the clinical relevance of C4 deposition in the kidney remains unclear.

Yan Yang et al. conducted a retrospective study on 642 patients with biopsy-confirmed IgAN, 41 of whom had C4 deposits in the mesangium in a biopsy [31]. In this group, lower serum albumin, higher proteinuria, and a higher rate of IgG, IgM and C1q deposition were observed. They identified glomerular C4 deposition and global sclerosis as independent risk factors for poor prognosis in IgAN [31].

## 7. TWEAK

**Figure 2:**
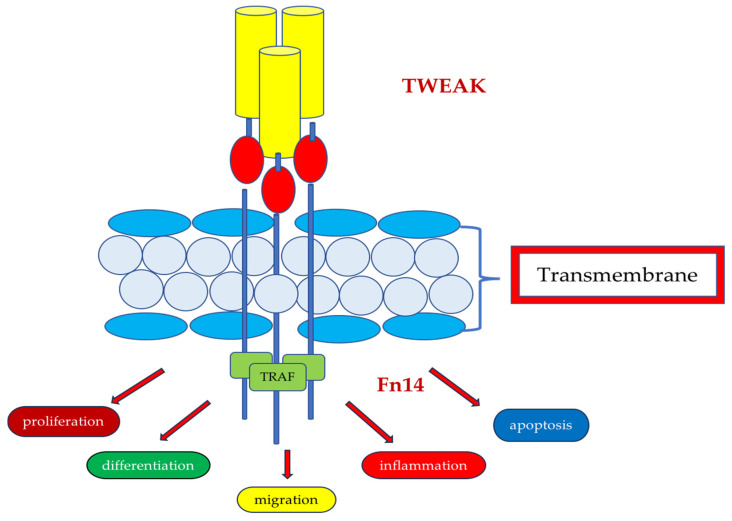
TWEAK/FN14—the TWEAK receptor, fibroblast growth factor-inducible-Fn14, is a type I transmembrane protein, the smallest member of the tumour necrosis factor (TNF) superfamily. The Fn14 intracellular domain contains a TNFR-associated factor (TRAF) binding site. By binding to the receptor, Fn14, TWEAK regulates cell proliferation, differentiation, migration, inflammation and apoptosis, acting as a mediator in chronic inflammatory processes.

TNF-like weak inducer of apoptosis (TWEAK), a member of the TNF superfamily of proteins, is a transmembrane type II glycoprotein circulating in plasma in a soluble form (Figure 2). TWEAK expression is widely distributed in human cells and tissues, including the kidneys [32]. By binding to the receptor, fibroblast growth factor-inducible-Fn14 (Fn14), TWEAK regulates cell proliferation, differentiation, migration, inflammation and apoptosis [32,33], acting as a mediator in chronic inflammatory processes, and may also play a role in the pathogenesis of renal failure [34].

Scientific evidence shows that urinary TWEAK concentration (uTWEAK, urinary TWEAK) correlates with, among others, lupus activity. These reports suggest a possible link between TWEAK expression, reflecting an active inflammatory process, and chronic kidney disease [35].

Sasaki Y et al. evaluated uTWEAK concentrations, proving that they were significantly higher in IgAN and other renal disease patients compared to healthy subjects [36]. In patients with IgAN, uTWEAK levels correlated remarkably with proteinuria and extra-capillary proliferation, indicating higher urinary concentrations of the molecule in patients with higher disease severity. Researchers also compared uTWEK concentrations of IgAN patients at diagnosis and follow-up, showing that urinary TWEAK concentrations in patients with clinical and partial disease remission were significantly reduced during follow-up. These data indicate the molecule’s potential for the minimal invasive control of the intensity of IgAN [36].

Similar conclusions were made by researchers Jin Sug Kim et al., who found that uTWEAK concentrations were significantly higher in patients with IgAN than in healthy people. uTWEAK levels correlated with the magnitude of proteinuria, which reflected the severity of inflammation in the kidney and histopathological classification, i.e., they were higher in patients with more advanced disease in the histopathological examination [34]. Currently, there are no studies on the serum and urine levels of TWEAK in paediatric IgAN patients, and this molecule seems to be a potentially important factor in the diagnosis and monitoring of inflammation progression and thus indirectly appears in response to treatment.

## 8. APRIL

The proteins that are part of the tumour necrosis factor (TNF) family induce pleiotropic cell responses, including growth, differentiation, and B-cell activating factor (BAFF) apoptosis, a B-cell activating factor that belongs to the TNF family of proteins that is expressed by T cells and dendritic cells [37].

Increased amounts of immunoglobulins were found in the supernatants of germinal centre-like B cells costimulated with BAFF, suggesting that BAFF plays an important role as a costimulator of B-cell proliferation and function.

BAFF is involved in the transmission of signalling receptors important in B cell development alongside another superordinate of the TNF family, A PRoliferation-Inducing Ligand (APRIL). APRIL, the central cytokine for B cell maturation and survival, is encoded by tumour necrosis factor ligand superfamily member 13 (TNFSF13) [37].

The GWAS study identified TNFSF13 as one of the genes associated with IgAN [38]. The available evidence indicated that immune activation by Toll-like receptor 9 (TLR9) is involved in the production of Gd-IgA. Makita Y et al. investigated how TLR9 activation in IgA-secreting cells causes the overproduction of nephritogenic IgA in an IgAN-susceptible ddY mouse strain and human IgA1-secreting cells [39].

Based on these findings, an injection of CpG-ODN (a ligand for TLR9) increased the production of abnormally glycosylated immune complexes (ICs) of IgA and IgG-IgA in ddY mice, leading to an exacerbation of renal damage. CpG-ODN-stimulated mice had elevated serum APRIL levels that correlated with levels of abnormally glycosylated IgA and IgG-IgA ICs. In vitro, the activation of TLR9 increased the production of nephritogenic IgA and APRIL in the spleen cells of ddY mice and human IgA1-secreting cells. Furthermore, APRIL-mediated TLR9-induced the overproduction of Gd-IgA1. These studies indicate that APRIL is a molecule that increases the production of Gd-IgA1 [39].

Kim YG et al. conducted a study in which mice with IgAN labelled “grouped ddY” (gddY) were administered intraperitoneally an anti-APRIL monoclonal antibody (anti-APRIL Ab) or control IgG (control Ab) twice weekly for 2 weeks [40]. The study was performed in mice in the early phase of IgAN (6–7 weeks). Significant decreases in albuminuria and tissue damage, serum IgA concentrations, and glomerular IgA deposition were observed in mice treated with anti-APRIL Ab [40].

Meyette JR et al. evaluated the pathogenic contribution of APRIL to IgAN and the therapeutic effect of neutralising a particle using an anti-APRIL monoclonal antibody in mice [41]. The administration of the APRIL antibody resulted in a lower serum IgA concentration, a decrease in circulating immune complexes, significantly reduced IgA, IgG and C3 deposition in kidneys, and decreased proteinuria compared to the control group [41].

In addition, researchers used, in a group of primates, VIS649—a highly potent humanised IgG2κ antibody against APRIL, which, through the unique involvement of epitopes, leads to the APRIL-mediated inhibition of B-cell activity. The use of VIS649 resulted in a dose-dependent reduction in serum IgA levels to 70% [41].

## 9. CD147

**Figure 3:**
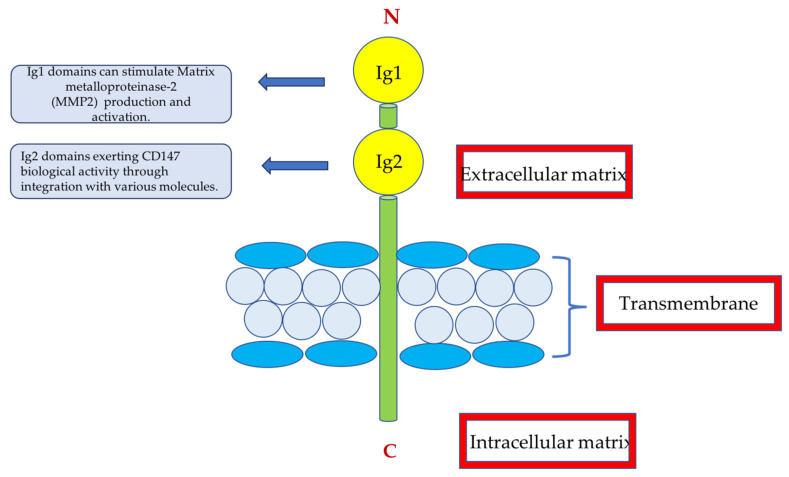
Cd147—a transmembrane glycoprotein composed of the intracellular (C-terminal), extramembrane (N-terminal) and transmembrane regions. The extracellular domain includes the Ig1 and Ig2 domains.

CD147 is a particle with great potential to study the active phase of glomerular damage in IgAN. It is an integral transmembrane glycoprotein of the immunoglobulin family, also known as extracellular matrix metalloproteinase inducer (EMMPRIN), first found in embryonic cancer cells (Figure 3). CD147 may also be present in the body in a soluble form (sCD147). The particle is widely distributed in human tissues, including the kidneys, liver, brain and spleen, and is involved in processes related to cell survival and migration [42].

In the kidneys, high CD147 expression is detectable in the basolateral side of tubular epithelial cells [43].

CD147 involvement in crucial processes of renal pathology has been described, for example, in lupus nephritis [44].

According to Mori Y et al., plasma CD147 levels correlate with eGFR in adults with inflammation-related renal diseases such as IgAN, IgA vasculitis, and diabetic nephropathy, indicating the progression of renal damage by inflammatory cell infiltration [45]. In addition, plasma CD147 concentrations correlate with the magnitude of changes in renal biopsy in these diseases, in particular IgAN [45].

Nagaya et al. investigated the contribution of CD147 in renal ischaemia and fibrosis by evaluating the particle concentration in the plasma and urine of patients with acute kidney injury (AKI) who underwent kidney biopsy [46].

In biopsy tissues in patients with acute tubulopathy (ATN), the induction of CD147 was observed in macrophages and lymphocytes in the injured mesenchymal region, but not in the injured tubules. Plasma and urine levels of CD147 were significantly increased in patients with ATN [46].

This study demonstrates the involvement of CD147 in processes in nephrological inflammatory diseases such as IgAN, and like most of the biomarkers discussed in the review, it has not been studied in the paediatric population.

## 10. Angiostatin

**Figure 4:**
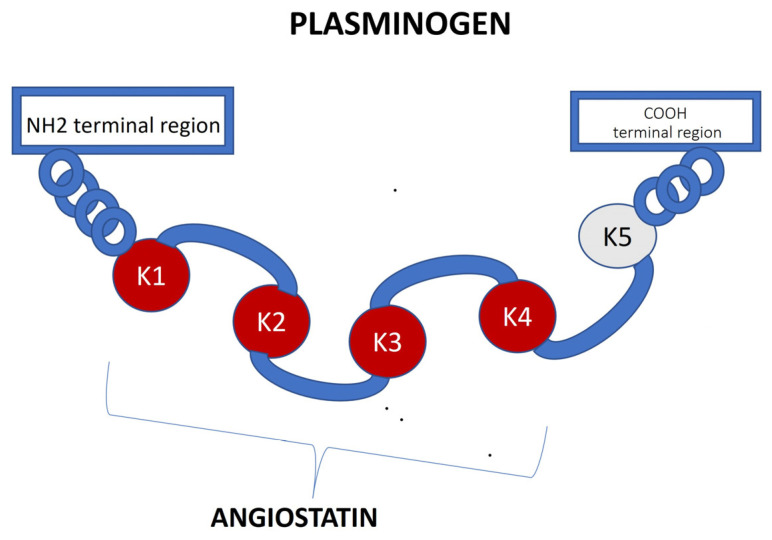
Angiostatin structure diagram—angiostatin is a 38 kDa proteolytic plasminogen fragment that inhibits angiogenesis in tumour cells. Matrix metalloproteinase 2 (MMP2) is an enzyme that releases angiostatin from plasminogen. Angiostatin blocks cell proliferation, induces apoptosis, inhibits endothelial cell migration and impairs the integrity of capillaries, thus leading to the inhibition of angiogenesis.

Angiostatin is a 38 kDa proteolytic plasminogen fragment that inhibits angiogenesis in tumour cells. Matrix metalloproteinase 2 (MMP2) is an enzyme that releases angiostatin from plasminogen (Figure 4) [47].

Angiostatin blocks cell proliferation, induces apoptosis, inhibits endothelial cell migration and impairs the integrity of capillaries, thus leading to the inhibition of angiogenesis [48].

Angiostatin also has anti-inflammatory effects by inhibiting leukocyte recruitment and neutrophil and macrophage migration [49].

Evidence indicates that angiogenesis-related factors, including angiostatin, play a role in the pathogenesis of advanced chronic kidney disease (CKD) [50]. An increased expression of angiostatin in the kidneys results in decreased renal capillary density and damage to the parenchyma [51].

Recently, the function of angiostatin as a potential marker in kidney disease has been investigated in, e.g., lupus nephropathy [52].

Tinafu Wu et al. evaluated urinary angiostatin levels in adult systemic lupus erythematosus (SLE) patients using an enzyme-linked immunosorbent assay (ELISA), demonstrating that these levels were significantly higher in the study group. In patients with SLE, urinary angiostatin was notably increased during the active stage of the disease in contrast to inactive SLE. Patients with the most advanced lupus nephritis (Class IV) had the highest levels of angiostatin in their urine. Urinary angiostatin concentrations strongly correlated with the rate of renal pathology persistence in SLE [53].

The function of angiostatin as a potential marker in lupus nephropathy in adults was also investigated by Chi Chiu Mok et al., showing that its urinary concentration correlated with increased proteinuria, i.e., increased inflammatory activity [54].

Recent proteomic studies of IgAN in adolescents and adults have found that urinary angiostatin levels correspond to the level of proteinuria, which is higher in patients with more advanced disease and increases with the stage of CKD.

A comparative analysis with the Oxford classification also revealed a correlation of urinary angiostatin levels with the severity of histopathological changes in renal biopsy, especially T1 (tubular atrophy/interstitial fibrosis), which may indicate an association with the chronic phase of inflammation. Adult patients with higher urinary angiostatin levels in the study group had an unfavourable prognosis [55].

These data show the promising role of angiostatin in the evaluation of stage of chronic lesions in IgAN, but there are no studies on paediatric patients.

## 11. Phosphatidylethanolamine Binding Protein-4 (PEBP4)

PEBP4 is a rarely investigated protein that potentially binds phosphatidylethanolamine and may act as a serine protease inhibitor. The overexpression of PEBP4 is implicated in tumour growth, progression and metastasis, suggesting a useful diagnostic marker for neoplasms. PEBP4 activation has also been shown to reduce the sensitivity of cancer cells to radiotherapy and chemotherapy by activating signalling pathways [56].

He P. et al. evaluated serum PEBP4 levels in CKD patients and found that they had elevated levels of this molecule, suggesting its utility as a diagnostic marker for CKD [56].

The role of PEBP4 in IgAN is unknown. Some studies conducted on patients with IgAN, in which serum and urine levels are assessed, indicate that an increased concentration of the molecule may be associated with a deterioration in kidney function.

Taylor et al. performed a proteomic analysis of urine samples and identified PEBP4 in IgAN patients [57]. Concentrations of this protein were significantly higher in patients with low eGFR, suggesting an association with CKD severity [57].

## 12. Summary

IgAN is the most common form of glomerulonephritis worldwide and is associated with an unfavourable prognosis.

The growing interest in recent years in the broad use of non-invasive factors in the diagnosis of IgAN has contributed to a significant development in knowledge in this field. Non-invasive investigations using these molecules as an alternative to renal biopsy have potential relevance for diagnosis, the determination of treatment efficacy and the assessment of disease activity. For the early screening of IgAN with isolated haematuria, these factors may be useful for early intervention and result in a reduction in the risk of impaired renal function.

In this review, studies on the potential application of the following biomarkers were analysed and presented above: Gd-IgA1, Anti-Gd-IgA1, IgA/C3, complement C4, TWEAK, APRIL, CD147, angiostatin, and PEBP4. The clinical studies discussed in this review underline that featured molecules show potential as biomarkers for the diagnosis and assessment of disease progress in IgAN (Table 1).

Of the biomarkers presented in the review, TWEAK and angiostatin are particularly worthy of attention. Several studies on the significance of these markers in adults with IgAN have been published in the past few years.

Urinary angiostatin is a potential marker of the chronic phase of inflammation in IgAN. The data stemming from the sources presented in this paper exhibit cases of inflated levels of this molecule in urine and emphasise the interconnection of these levels with the magnitude of proteinuria.

A similar interconnection was indicated by researchers with regard to uTWEAK. Concentrations of this molecule were significantly higher in patients with IgAN than in healthy controls and uTWEAK levels in IgAN patients correlated with the severity of proteinuria.

For the diagnosis of IgAN and to predict prognosis, urinary biomarkers seem to have an advantage over serum biomarkers because they are more specific to the kidneys.

There are several limitations for studies in the usage of biomarkers in the diagnosis of IgAN. Although we exhibited that the data stemming from the sources presented in this paper prove that these novel markers correlated with proteinuria and disease activity, their role in the assessment of disease progression in IgAN in children is still unclear. Moreover, these particles were evaluated mainly in studies with adults and require further studies, so as to have the prospect of being beneficial for the cases of children.

Therefore, in order to be able to routinely and extensively measure them in this group of patients, further research is needed to determine both the reference range and the factors contributing to changes in markers’ levels in the paediatric population.

Further prospective studies will provide more information on the performance of these markers in IgAN in children compared to conventional markers and renal biopsy.

## Figures and Tables

**Table 1:** Analysed biomarkers.

Biomarker	Serum/Plasma	Urine	References
GdIgA1	IgAN > healthy control		[25]
	IgAN > other kidney diseases		[20]
	In IgAN associated with a risk of disease activity		[19]
	Potential marker of IgAN progression in children		[21]
Anti-GdIgA1	IgAN > healthy control		[22,23]
	IgAN > other kidney diseases		[22,23]
	In IgAN related to severity of proteinuria		[18]
IgA/C3	IgAN > other kidney diseases		[27]
	Unrelated to proteinuria and GFR		[27,28]
C4 complement	In IgAN related to severity of proteinuria and GFR		[30]
	Glomerular C4 deposition—risk factor for poor prognosis in IgAN		[31]
TWEAK		IgAN and other renal diseases > healthy control	[34,36]
		In SLE related to disease activity	[35]
		In IgAN related to severity of proteinuria	[34,36]
APRIL	Administrated APRIL antibody related to lower GdIgA1 in mice		[40,41]
CD147	In IgAN and other inflammation-related renal diseases related to GFR		[45]
	Increased in ATN	Increased in ATN	[46]
Angiostatin		In IgAN and SLE related to severity of proteinuria	[53,55]
		In SLE > healthy control?	[53]
		Potential marker of chronic phase of inflammation in IgAN	[55]
PEBP4	Elevated level in patients with CKD		[56]
	In IgAN related to GFR		[57]

Gd-IgA1—galactose-deficient iga1, Anti-Gd-IgA1—antibodies directed against galactose-deficient IgA1, TWEAK—TNF-like weak inducer of apoptosis, APRIL—A PRoliferation-Inducing Ligand, CD147ATN—acute tubulopathy, PEBP4—phosphatidylethanolamine binding protein-4, CKD—chronic kidney disease.
